# Implementation of an Online Reporting System to Identify Unprofessional Behaviors and Mistreatment Directed at Trainees at an Academic Medical Center

**DOI:** 10.1001/jamanetworkopen.2022.44661

**Published:** 2022-12-02

**Authors:** I. Michael Leitman, David Muller, Sophie Miller, Basil G. Hanss, Thomas F. Catron, William O. Cooper, Marta Filizola

**Affiliations:** 1 Medical Education and Surgery, Graduate Medical Education, Icahn School of Medicine at Mount Sinai, New York, New York; 2 Medical Education and Medicine, Medical Education, Icahn School of Medicine at Mount Sinai, New York, New York; 3 Graduate School of Biomedical Sciences, Icahn School of Medicine at Mount Sinai, New York, New York; 4 Department of Medicine, Icahn School of Medicine at Mount Sinai, New York, New York; 5 Department of Medical Education, Vanderbilt Center for Patient and Professional Advocacy, Vanderbilt University Medical Center, Nashville, Tennessee; 6 Pediatrics and Health Policy, Faculty Affairs, Vanderbilt University School of Medicine, Nashville, Tennessee; 7 Department of Pharmacological Sciences, Icahn School of Medicine at Mount

## Abstract

**Importance:**

Unprofessional behaviors and mistreatment directed at trainees continue to challenge the learning environment. Academic medical institutions should encourage reports of inappropriate behavior and address such reports directly to create a safe learning environment.

**Objective:**

To determine the feasibility of creating and implementing an online reporting system for receiving and reviewing complaints of unprofessional behavior directed toward or experienced by students, postdoctoral trainees, and residents.

**Design, Setting, and Participants:**

This cohort study assessed implementation of an online reporting system (feedback form) with a method for triaging reports, providing both positive and negative feedback, as well as adjudication and transparent public disclosure of aggregate data. The system was launched at a large urban academic medical center with numerous trainees that is fully integrated with a health system of 8 hospitals. Participants included faculty who interact with trainees, medical students, graduate students and postdoctoral fellows, and residents and clinical fellows. Follow-up began in October 2019 (at the time of tool launch) and lasted through December 2021. Data were analyzed from January to March 2022.

**Main Outcomes and Measures:**

The primary outcomes were the numbers and types of reports according to the reporter and the person reported about.

**Results:**

Participants included 2900 faculty who interact with trainees, 600 medical students, more than 1000 graduate students and postdoctoral fellows, and 2600 residents and clinical fellows. Trainees submitted 196 reports, 173 (88.3%) of which described unprofessional interactions. Among the reports describing unprofessional behavior, 60 (34.7%) were from medical students, 96 (55.5%) were from residents and fellows, 17 (9.8%) were from graduate students or postdoctoral trainees, and 78 (45.1%) were from men. The majority of negative reports described behaviors by faculty (106 [61.3%]), followed by residents and fellows (24 [13.9%]). Twenty faculty (<1.0%) accounted for 52 (50.0%) of the 104 reports describing unprofessional behaviors. Since implementation, most trainees are aware of this process. An increasing number have reported instances of mistreatment, and those who shared concerns through the online system report satisfaction with the outcome of the response to the report.

**Conclusions and Relevance:**

In this cohort study, the new reporting mechanism facilitated identification of the small number of individuals associated with unprofessional behaviors toward trainees and increased awareness of the school’s commitment to creating a safe learning environment.

## Key Points

**Question:**

Can a structured reporting system be adopted by trainees at all levels in a large academic medical center?

**Findings:**

In this cohort study, learners became familiar and comfortable reporting mistreatment in an environment when they were assured that all such reports are reviewed and handled in a confidential manner.

**Meaning:**

These findings suggest that providing an organized infrastructure and sharing global data is a feasible intervention to help mitigate unprofessional behaviors directed at trainees.

## Teaser

This cohort study evaluates the implementation of an online reporting system to identify unprofessional behaviors and mistreatment directed at trainees at an academic medical center.

## Introduction

Professionalism in medicine has been described over the course of several decades and includes key precepts such as communication, knowledge, technical skills, clinical reasoning, emotions, values, and reflection in daily practice.^1,2^ The focus of professionalism has typically been on clinical practice and the importance of extending these principles to physicians-in-training.^3^ As professionalism in science has typically referred to scientific integrity, objectivity, clarity, reproducibility, and clear communication when reporting and applying the results of scientific activities, less attention has been paid to interpersonal interactions and mentee emotions, values, and reflections, despite being equally important for scientists and scientists-in-training. Similarly, unprofessional behavior, including mistreatment, has been described extensively in medicine, but less so in biomedical research.^4,5^

Establishing an environment that is free of mistreatment and unprofessional behaviors in all settings is arguably the most challenging and most important priority across all missions in an academic medical center. Acknowledging, reporting, addressing, and, over time, reducing these behaviors helps establish a culture of justice, equity, and inclusion that leads to greater diversity, creativity, safety, and productivity.

This study is a feasibility study and describes the efforts of a large urban academic medical center, including a medical school, a graduate school of biomedical sciences, and a health system, to design, implement, and sustain a model of professional accountability that encompasses all aspects of education and training. Before embarking on the development of this system, we did an exhaustive search of the literature to identify a portal that might be suitable for this purpose but were unable to identify one.

## Methods

### Setting

In designing the follow-up of the cohort of individuals who received reports, we followed the Strengthening the Reporting of Observational Studies in Epidemiology (STROBE) reporting guideline for cohort studies.^6^ The institutional review board at the Icahn School of Medicine at Mount Sinai (ISMMS) reviewed the protocol and the data collection and made the determination that the study did not qualify as human participants research and waived the need for informed consent in accordance with 45 CFR §46.

Mount Sinai Health System is an urban academic medical center in New York City that includes the ISMMS, 8 teaching hospitals, more than 7000 faculty, more than 400 ambulatory practices, and more than 45 000 employees. ISMMS has an undergraduate medical education (UME) program (600 students, including 95 dual degree MD/PhD students in partnership with the graduate school), a graduate school of biomedical sciences (more than 230 PhD students in 3 different programs, more than 500 master’s students, and 540 postdoctoral fellows in more than 300 research laboratories), and a graduate medical education (GME) consortium with more than 2600 residents and fellows.

The initial motivation to critically review and revise the school’s approach to mistreatment and unprofessional behaviors arose from analysis of data collected by the UME and GME programs as part of a review of accreditation requirements, as well as concerns about well-being, equity, and racism. The UME program was struggling to respond to several years of Association of American Medical Colleges (AAMC) Graduation Questionnaire data that revealed greater than national average mistreatment incidents directed at trainees, lower than national average reporting and awareness of policies, and fear of retaliation. Similarly, residents and fellows expressed concerns to GME leadership about mistreatment in the clinical learning environment and fear of retaliation that were contributing to burnout. There were sparse data to support concerns about the climate for PhD and master’s degree students, as well as postdoctoral trainees, but there were similar anecdotal reports about the experience of learners in these settings. At the same time, increasing attention was being paid nationally to mistreatment of graduate students and postdoctoral trainees.^7^

### Developing the Reporting Tool

The online reporting tool and process were implemented by an interprofessional group of the institution’s key stakeholder groups. Several elements were critically important in drafting a policy and developing an online tool for reporting mistreatment and unprofessional behavior. It had to be a single, central repository universally applicable to all learners; easily accessible; easy to navigate; anonymous, with an option to identify oneself; include a universally accepted definition of mistreatment behaviors; the ability to report positive behaviors and accolades; the ability to track data, report trends, and be searchable; the ability to send automatic notifications to responders, thus enabling a timely response; the ability to annotate responses and actions; and offer options for delayed reporting to engender a semblance of control and mitigate fear of retaliation for the reporter.

The review of and response to reports had to be accountable to an individual or small group of individuals whose adjudication would, over time, be increasingly valid, reliable, and consistent. Every response or intervention had to be timely and include a reminder of the institution’s strong policies against retaliation. The response needed to include closing the loop with individual reporters who identified themselves by notifying them that the case was triaged and reviewed, and with the school’s community at large through regular updates. Finally, the policy, online tool, and protocol had to be acceptable to multiple stakeholders, including institutional leadership; legal counsel; human resources; medical school leadership; the institution’s ombuds office; Title IX Coordinator; the offices for diversity, well-being, and gender equity; students; and trainees.

After receiving approval from the dean of ISMMS to undertake this initiative, representatives from each of these groups met regularly over the course of 12 months. These individuals would later constitute the committee that would oversee the program, meeting quarterly to review data and continuously refine the process.

The group identified several important concerns that guided their planning: How was retaliation going to be meaningfully addressed? Was the policy going to be meaningful and support interventions even when the persons described as exhibiting unprofessional behavior were prominent, senior, or highly productive faculty or administrators? Would all the data tracked by this tool be discoverable and create unacceptable legal risk for the institution? Would the process be truly confidential? How, how often, and with whom should data be shared? How would training be made available to everyone who needed to distinguish between providing feedback, offering remediation, formally investigating, and referring to appropriate authorities as required by law, regulation, or policy (ie, Title IX office, ombuds, or human resources)?

There was much discussion about including “publicly embarrassed” in the definition of mistreatment. Did that include not knowing the answer to a clinical management question that a surgeon might ask a student in the operating room, surrounded by nurses, residents, fellows, and other students? Did it include probing scientific questions from a graduate student’s thesis committee? Were these examples of appropriate teaching and learning, or could they be distinguished from intentional public humiliation, which might be subjective depending on the perspective of the persons involved?

Although there was consensus that the existing policy and protocol needed updating and clarification, these concerns led to resistance from some stakeholders to key elements such as using explicit language (eg, “mistreatment”), maintaining an online database of reports, and sharing aggregate data with the community of students, trainees, faculty, and leadership. In an effort to overcome this resistance, the policy used the term “mistreatment and unprofessional behaviors” instead of exclusively using the term “mistreatment.”

### Gaining Clarity

In creating the reporting tool, the group used the AAMC Graduation Questionnaire (GQ) definition of mistreatment (eAppendix 1 in the Supplement). We adjusted GQ questions specifically related to professional behaviors in the clinical learning environment because of the priority of making the tool universally applicable to graduate school students and postdoctoral trainees (eAppendix 2 in the Supplement). Technical aspects of the tool and an image of the online reporting form are provided in eAppendix 3 and eAppendix 4 in the Supplement.

### Addressing Retaliation

To address concerns about retaliation, an explicit statement about retaliation and the institution’s commitment to address it was included in the policy. In addition, everyone receiving feedback for a reported mistreatment behavior would be reminded of the institution’s zero tolerance approach to retaliation. Reporters could request delayed action and specify the length of the delay; they could request that the report be acted upon only if other reports on that same person existed; and they could request that the person reported be monitored and the incident not acted upon. Exceptions to requested delays occurred if an incident required mandatory reporting (eg, discrimination or sexual harassment).

### Recognizing Positive Behaviors

Although the purpose of this work was addressing negative behaviors, the group also recognized that students and trainees more commonly experience positive and admirable behaviors from outstanding role models. It was important to provide a venue for sharing these accolades. Making them part of the feedback form (and not calling it a mistreatment form) allowed the group to address concerns that this reporting mechanism would reflect unfairly on the institution, staff, and faculty. Faculty who received an accolade would be contacted immediately, and the report shared with them and their department chairs.

### Launch

Once there was consensus among members of the group drafting the policy and protocol, the documents were shared with the dean of ISMMS for approval. Communication across all key stakeholder groups included presentations at department meetings and grand rounds, presentations at standing leadership meetings with chairs and deans, and incorporation into orientation for students and postdoctoral trainees. Links for reporting were made available on hospital workstations and on school websites. Upon receipt, reports were triaged by the respective dean within 24 hours, who then determined the course of action. Reports that were either egregious or revealed patterns of behavior were escalated to remediation, formal investigation, and/or disciplinary action by trained human resource personnel.

### Statistical Analysis

The analysis includes the date the online reporting tool went live in late 2019 through December 2021. A linear trend model using analysis of variance was created for the percentage difference in the moving mean number reports per quarter from the beginning through the end of follow-up using Tableau Desktop software version 2022.2.1 (Tableau Software). Although there were occasional reports regarding staff behavior, which were addressed using existing human resources practices, the focus of the frequency of reports was on the cohort of faculty who interact with students and trainees, as well as the students and trainees themselves. Data were analyzed from January to March 2022.

## Results

**Figure 1.**
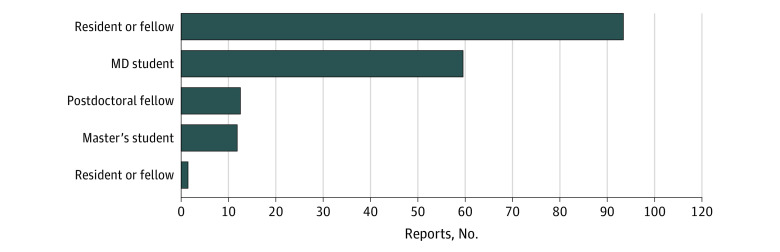
Feedback Reports by Trainee Role Percentage of reports, number of reports, and reporter role for data collected from October 25, 2019, to December 31, 2021.

**Figure 2.**
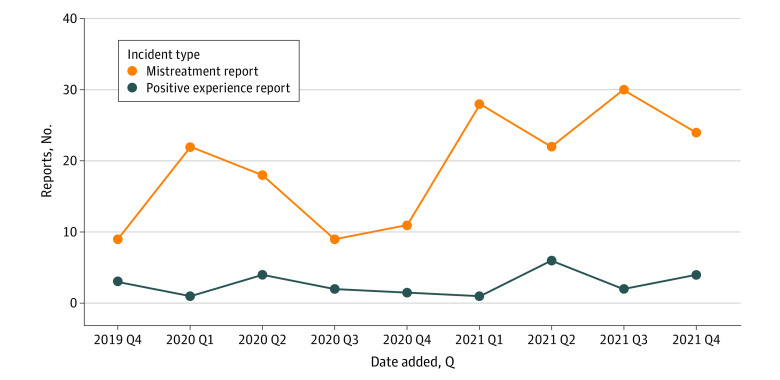
Timeline of Feedback Reports Received Timeline of reports received from October 25, 2019, to December 31, 2021. Q indicates quarter.

There were a total of 196 reports by medical students, graduate students, residents, fellows, and postdoctoral trainees, with residents and fellows being the most frequently contributing category of trainees (Figure 1). A total of 173 (88.3%) of the reports described unprofessional interactions. As shown in Figure 2, the use of the online tool increased over time. Although 69 reporters (39.9%) provided their identity in submitting negative feedback reports, a larger number of them (104 reporters [60.1%]) submitted their reports anonymously. On the other hand, 14 (60.9%) positive experience reports were signed, and a smaller number were submitted anonymously (9 reports [39.1%]). The relationship between the reporter and the role of the person being reported is listed in Table 1 and by sex in Table 2. The majority of negative feedback reports described faculty behaviors (106 [61.3%]), followed by residents and fellows (24 [13.9%]). Among the reports describing unprofessional behavior, 60 (34.7%) were from medical students, 96 (55.5%) were from residents and fellows, and 17 (9.8%) were from graduate students or postdoctoral trainees.

**Table 1.** Number of Negative Feedback Reports by Reporter Role vs Role of the Person Being Reported

Reporter role and role in institution of the person who was reported	Reports, No. (%) (N = 173)
Master’s student	
Faculty	1 (0.6)
Master's student	1 (0.6)
MD student	
Administrator	3 (1.7)
Chief resident	1 (0.6)
Faculty	25 (14.5)
Fellow	5 (2.9)
MD student	8 (4.6)
MD and PhD student	1 (0.6)
Nurse	1 (0.6)
Other^a^	5 (2.9)
Patient	1 (0.6)
PhD student	3 (1.7)
Postdoctoral fellow	1 (0.6)
Resident	6 (3.5)
PhD student	
Faculty	2 (1.2)
Postdoctoral fellow	
Administrator	1 (0.6)
Faculty	12 (6.9)
Resident or fellow	
Administrator	4 (2.3)
Chief resident	2 (1.2)
Faculty	66 (38.2)
Fellow	4 (2.3)
Nurse	6 (3.5)
Other	8 (4.6)
PhD student	1 (0.6)
Resident	5 (2.9)

^a^ Other indicates adminstrative staff, physican assistants, locum tenens, and non-degree students.

**Table 2.** Negative Feedback Reports by Sex

Sex of reporter	Sex of person being reported, No. of individuals		
	Female	Male	Unknown
Female	29	46	4
Male	23	32	2
Unknown	11	9	17

The most common negative feedback reported was (1) publicly embarrassed or humiliated (95 reports [54.9%]); (2) subjected to offensive remarks related to gender, sexual orientation, national origin, race, color, religion, or any other category protected by law (57 reports [32.9%]); and (3) denied opportunities for training or rewards on the basis of membership in a protected group (16 reports [9.2%]). In contrast, most positive experiences highlighted people who (1) create an environment that values diversity, equity, and inclusion (15 reports [65.2%]), (2) demonstrate exemplary collaboration (13 reports [56.5%]), and/or (3) treat others with dignity and respect (17 reports [73.9%]).

**Figure 3.**
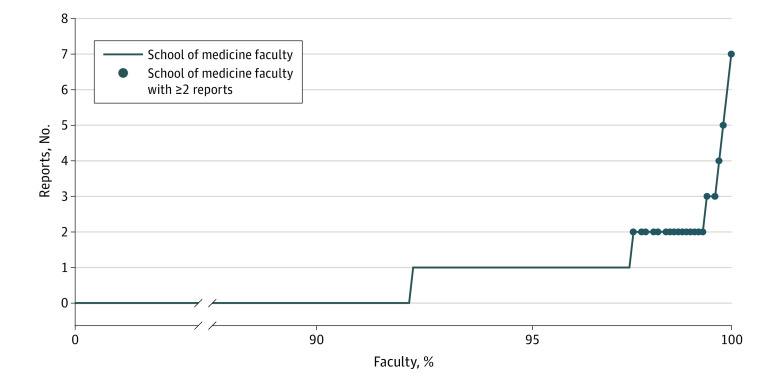
Distribution of Reports by Faculty Distribution of reports describing unprofessional behaviors of faculty toward trainees in an academic medical center from October 25, 2019, to October 31, 2021.

The majority of negative feedback reports described faculty behaviors (61.3%). Although more than 90% of the 2900 faculty who interact with trainees did not receive any reports describing unprofessional behaviors, 20 faculty (<1.0%) accounted for half of the reports describing faculty unprofessional behavior (Figure 3). A total of 163 reports (94.0%) were handled by a single discussion with the subject of the report; 10 reports (6.0%) were escalated to an intervention that included a written warning or modification of faculty duties. A total of 16 reported faculty (10.0%) received more than 1 negative report. A total of 14 reported faculty (8.0%) were referred for a physician wellness evaluation.

In the 2021 GQ, 143 medical students (100%) reported being aware of the policy compared with 97.9% of students nationally. A total of 140 (96.7%) knew the procedures for reporting (compared with 91.1% of medical students nationally), 50 (35.0%) indicated that they had experienced mistreatment behaviors (compared with 40.3% of medical students nationally), 51 (35.7%) indicated that they had reported these behaviors (compared with 27.3% of medical students nationally), and 47 (33.3%) reported that they were satisfied or very satisfied with the outcome of reporting (compared to 46.3% of medical students nationally). These results represent modest increases over the past 5 years. Satisfaction with the outcome of having reported these behaviors also improved modestly but remains below the national average. More than 90% of residents and fellows (1596 individuals) reported awareness of the reporting mechanism.

### Ongoing Review and Refinements

Several gaps became evident in the first year following the launch of the feedback form. There was no reliable system in place for automatically identifying people who mistreated across multiple areas. For example, the trend created by separate reports from a student, resident, or postdoctoral trainee about bullying behavior by the same faculty member whose work bridged the clinic and the laboratory could go unnoticed, unless it was picked up manually and someone asked for a report from the database.

Even more disconnected were reports about the same person that might have been submitted by a nurse, faculty colleague, or member of the administrative staff using entirely different reporting mechanisms. As an interim solution for the clinical environment, the head of human resources, chief medical officer, and deans for GME and UME agreed to meet monthly and compare notes about concerning incidents, departments with clusters of reports, and potential trends. Data obtained from surveys indicate that more than 90% of residents and clinical fellows are aware of how to report using the portal.

Because of initiatives the medical center had undertaken regarding addressing professionalism in the clinical space, several improvements were made in the protocol and process. First, centralizing patient and coworker observation data permitted a searchable database to cross-reference and research potential patterns of unprofessional behavior. Second, training peer messengers to deliver observations and awareness to colleagues identified in reports promoted consistency and reliability in messaging. Third, identifying a small contingent of stakeholder leaders to review and identify action steps for reports of egregious behavior (eg, sexual boundary violation, harassment, intoxication, and violence) ensured that the institutional response was coordinated and that the safety of reporter and colleague was addressed. Finally, there was growing recognition of the importance of working closely with institutional leadership to contend with prominent faculty or staff who had been reported for mistreatment.

## Discussion

In this cohort study, by providing a secure protocol for reporting and addressing mistreatment and unprofessional behavior, student and trainee responses to periodic surveys suggest an increasing awareness and use of reporting mechanisms to describe unprofessional behaviors in the learning environment. The protocol also bridges the UME and GME continuum as well as the learning and research environments of the Graduate School of Biomedical Sciences at ISMMS. Feedback and other interventions occur in real time, including contacting reporters who self-identify and quarterly sharing of data with the entire academic medical center community.

We managed to overcome initial substantial resistance to explicitly acknowledging and addressing mistreatment and unprofessional behaviors directed at trainees. We continue to be challenged by a perception, particularly among some students, arguably more so among graduate students as well as postdoctoral trainees, that the system is inaccessible, that nothing will be done, and that it is not safe to report these behaviors. Concerns about retaliation and psychological safety might discourage reporting.

Genuine culture change requires buy-in from all stakeholders.^8^ Endorsement from the dean of ISMMS and the leadership group of deans and chairs, combined with ample representation by students and trainees in every step of development and implementation, has allowed us to begin shifting the mental models that are at the heart of historical inertia related to mistreatment.

### Lessons Learned

Navigating the process of securing buy-in, refining policies, establishing a reporting and feedback system required an iterative, time-consuming approach, with extensive consensus building, compromises, and course correction along the way. Implementation required a substantial investment in reaching agreement around every aspect of this undertaking, from agreeing that there was a need for a policy and protocol, to the language used and details of execution, to vetting the people who should be directly involved in reviewing and intervening. Human resources was worried that we would not know how to assess egregious incidents, legal counsel was worried that we could not protect these data from discovery, and students and trainees were worried that we would neither be completely transparent in our actions nor accountable to our constituents. Among the faculty, there were a handful of early adopters, many others who have come to appreciate this work over the course of the past 2 years, and others who continue to believe that we are undermining academic rigor and clinical excellence by catering to the needs of “overly sensitive” students and trainees. The results of the GQ survey after implementation have provided reinforcement to institutional leaders about the importance of creating a safe platform and process for reporting unprofessional behaviors in the learning environment.

### Limitations

There remain several limitations to this initiative. We do not have a formal and systematic method for tracking patterns of behavior reported by different learners and trainees, across academic departments, including events that occur between the health system and school of medicine. This will remain a challenge as long as there are multiple mechanisms for reporting mistreatment and unprofessional behavior that are not integrated. In addition, the program was implemented in a single institution in an urban environment. The measures of student satisfaction with reporting were extrapolated from GQ questionnaire results as we did not directly survey students before and after implementation of the online tool and process, which would have also facilitated an understanding of whether students were more willing to report behaviors after implementation. Although the tool and process were implemented in a large academic setting with a network of different service and faculty types, whether the findings from this work will generalize to different organizations in different settings is unknown but testable. Although we believe that the increase in reports reflects general understanding and acceptance of the program, we cannot state unequivocally that this reflects a general change in climate, though we remain hopeful. We continue to monitor surveys of stakeholders to determine the impact on our culture.

## Conclusions

By role modeling professional behaviors, providing timely and consistent feedback when unprofessional behavior occurs, and transparently sharing mistreatment data and outcomes with trainee communities, we were able to feasibly implement an online tool and response system for learners to share reports of both professional and unprofessional behavior in an academic medical center. Although our policy and protocol have begun to untangle the complex web of unprofessional and mistreatment behaviors that are all too common in academic medicine, it will take time to appreciate their long-term impact on the underlying culture as we continue to enhance our learning environment.

## Notes

Supplement. **eAppendix 1.** Association of American Medical Colleges (AAMC) List of Medical Student Mistreatment Behaviors **eAppendix 2.** Expected Professional Behaviors in the Clinic and Laboratory **eAppendix 3.** Technical Aspects of the System **eAppendix 4.** Experience Reporting Form
eReferences.
