# Supplementary material for: Implementation of an Online Reporting System to Identify Unprofessional Behaviors and Mistreatment Directed at Trainees at an Academic Medical Center
Source: JAMA Netw Open. 2022 Dec 2;5(12):e2244661. doi: 10.1001/jamanetworkopen.2022.44661 (PMC9719046; doi:10.1001/jamanetworkopen.2022.44661)
Supplement: Supplement. — eAppendix 1. Association of American Medical Colleges (AAMC) List of Medical Student Mistreatment Behaviors eAppendix 2. Expected Professional Behaviors in the Clinic and Laboratory eAppendix 3. Technical Aspects of the System eAppendix 4. Experience Reporting Form eReferences. [file jamanetwopen-e2244661-s001.pdf]

Supplemental Online Content

Leitman IM, Muller D, Miller S, et al. Implementation of an online reporting system to identify unprofessional behaviors and mistreatment directed at trainees at an academic medical center. *JAMA Netw Open*. 2022;5(12):e2244661. doi:10.1001/jamanetworkopen.2022.44661

eAppendix 1. Association of American Medical Colleges (AAMC) List of Medical Student Mistreatment Behaviors

eAppendix 2. Expected Professional Behaviors in the Clinic and Laboratory

eAppendix 3. Technical Aspects of the System

eAppendix 4. Experience Reporting Form

eReferences.

This supplemental material has been provided by the authors to give readers additional information about their work.

© 2022 Leitman IM et al. *JAMA Network Open.*

eAppendix 1. Association of American Medical Colleges (AAMC) List of Medical Student Mistreatment Behaviors[1]

Threatened with physical harm or physically harmed
Required to perform personal services
Subjected to offensive remarks related to gender, sexual orientation, national origin, race, color, religion, or any other category protected by law
Denied opportunities for training or rewards based upon membership in a protected group
Subjected to lower evaluations or grades solely because of membership in a protected group
Publicly embarrassed or humiliated
Subjected to unwanted sexual advances
Asked to exchange sexual favors for grades or other rewards

https://www.aamc.org/media/23541/download

© 2022 Leitman IM et al. *JAMA Network Open.*

eAppendix 2. Expected Professional Behaviors in the Clinic and Laboratory (modified[1,2])

**Professionalism in Clinical Settings**	**Professionalism in Laboratory Settings**
Respecting patient confidentiality	Respecting confidentiality
Showing respectful interaction with students	Showing respectful interaction with students
Being respectful of patients' dignity and autonomy	Being respectful of other researchers' dignity and autonomy
Respecting diversity	Respecting diversity
Using professional language/ avoiding derogatory language	Using professional language/ avoiding derogatory language
Being respectful of house staff and other physicians	Being respectful of other lab personnel, trainees, and investigators
Showing empathy and compassion	Showing empathy and compassion
Resolving conflicts in ways that respect the dignity of all involved	Resolving conflicts in ways that respect the dignity of all involved
Actively listened and showed interest in patients	Actively listened and showed interest in other researchers
Being respectful of other health professions	Being respectful of other science professions
Advocating appropriately on behalf of patients	Advocating appropriately on behalf of other scientists
Taking time and effort to explain information to patients	Taking time and effort to explain information
Providing direction and constructive feedback	Providing direction and constructive feedback
Being respectful of other specialties	Being respectful of other scientific specialties

© 2022 Leitman IM et al. *JAMA Network Open.*

eAppendix 3. Technical Aspects of the System

The needs and rationale detailed above informed all aspects of the reporting tool, from the user interface to the data structure. A Feedback Form, hosted on Formstack.com (Fishers, IN), handles initial data collection. Data are routed and stored in cloud-based relational databases via Airtable ™ (San Francisco, CA), customized to enable collaboration while emphasizing data security.

The Feedback Form (Appendix) allows for anonymous or identifiable reports of mistreatment and unprofessional behaviors, positive experiences, and learning experiences. All submission types require the reporter's role and selection of the type of report; this informs branching logic within the form and routing logic following submission. All reporters may opt to provide their name, email, gender, and the date of the event. If the reporter chooses to submit their email, they receive a confirmation following submission.

Mistreatment and *Positive experience* reports require the input of the name and role of the person they are reporting. We optionally prompt for the department and gender of the person they are reporting. Although voluntary, this information helps inform accurate attribution and appropriate action following submission.

If the reporter selects *Mistreatment Report* from the list of report types, the branching logic shows relevant fields. In developing this form component, we emphasized reporting mechanisms that foster the safety of the reporter, including options for delayed action. We offered three delay request options: request delayed action, request action only if similar reports about the same person exist, request monitoring with no action. We prompt the reporter to provide their involvement in the experience (directly involved or bystander), the incident type(s) (eTable 3), and the description of their experience. The reporter will then indicate if the incident was previously reported. If *yes* (the incident was previously reported), the reporter chooses or inputs where the experience was reported, and who addressed the report. If applicable, the reporter can upload a previous report and provide the subsequent intervention. If *no* (the incident was not previously reported), the reporter can submit the form. If the reporter selects *Positive Experience Report*, relevant fields appear. The reporter chooses from a list of positive report types (eTable 4) and enters a description of their experience. If the reporter selects *Learning Experience Report*, relevant fields appear. The reporter indicates if feedback is positive or negative, inputs the educator's name, course name, and provides a description of the experience. Course names are automatically suggested from a repository of active courses.

Based on the reporter type and report type, the appropriate parties receive a notification linked to the Feedback Form database. Learning Experience reports route to an independent database.

Role-based authentication secures the central database containing mistreatment and positive experience reports. Users with specific permissions can access, read, comment, and write directly to the database record. Users with write permissions can append notes on intervention and outcomes within the record. The database stores record-specific activity logs. Metadata in the activity log includes the last user, last modified time, and specific modification details.

Adding and manipulating data directly within the database encourages data quality and integrity while supporting needed flexibility.

Data from the feedback form database syncs with read-only reporting databases, separated by Department. The reporting databases enable staff with explicit permissions to view redacted reports and assist with aggregating quarterly reports while maintaining anonymity and data integrity.

© 2022 Leitman IM et al. *JAMA Network Open.*

eAppendix 4. Experience Reporting Form

Mount Sinai

ISMMS Feedback Form

Reporter name (optional)

First Name | Last Name

Reporter Email

Reporter role*

Reporter gender

Select type of report*

Save and Resume Later

Submit Form

Select type of report*

Mistreatment Report

Name of the person you're reporting*

First Name | Last Name

Role in institution of the person you're reporting*

Department of the person you're reporting

Gender of the person you're reporting

Mistreatment Report

Date of the event (optional)

02 | 02 | 22

Request delayed action?
○ Yes ● No

Request action only if other reports on the same person exist?
○ Yes ● No

Request monitoring with no action?
○ Yes ● No

What is your involvement in this experience?

Directly Involved

Incident type

- ☐ Threatened with physical harm or physically harmed
- ☐ Required to perform personal services
- ☐ Subjected to offensive remarks related to gender, sexual orientation, national origin, race, color, religion, or any other category protected by law
- ☐ Denied opportunities for training or rewards based upon membership in a protected group
- ☐ Subjected to lower evaluations or grades solely because of membership in a protected group
- ☐ Publicly embarrassed or humiliated
- ☐ Subjected to unwanted sexual advances
- ☐ Asked to exchange sexual favors for grades or other rewards
- ☐ Subjected to the threat of revoking visa status for foreign nationals

Description of Experience*

© 2022 Leitman IM et al. *JAMA Network Open.*

Reporting information

Was this previously reported?

○ Yes ○ No

Where was this experience reported?

○ Site Director
○ Clerkship Director
○ Chief Resident
○ Attending Physician
○ PI
○ Advisor
○ Ombudsperson
○ Student mistreatment Committee
○ Program Director
○ Office Director
○ Chairman
○ Course Director
○ Dean
○ Student Affairs (Medical Education)
○ Student Affairs (Graduate Education)
○ Title IX Coordinator
○ Other:

Who addressed this report?

First Name | Last Name

Upload Previous report (if applicable)

Choose File **Remove File** No File Chosen

optional

If applicable, what was the intervention?

Submit Form

Select type of report*

Learning Experience Report

Learning Experience Report

Date of the event (optional)

02 02 22

Positive or Negative Feedback?*

○ Positive ○ Negative

Educator Name (optional)

First Name | Last Name

Course Name*

Description of Experience

Save and Resume Later

Submit Form

Privacy - Terms

© 2022 Leitman IM et al. *JAMA Network Open.*

Select type of report*

Positive Experience Report

Name of the person you're reporting*

First Name

Last Name

Role in institution of the person you're reporting*

Department of the person you're reporting

Gender of the person you're reporting

Positive Experience Report

Date of the event (optional)

02 02 22

Positive Feedback Type

- ☐ Treats others with dignity and respect
- ☐ Demonstrates exemplary collaboration
- ☐ Creates an environment that values diversity, equity and inclusion
- ☐ Demonstrates concern for student/trainee wellness
- ☐ Outstanding (check all that apply)

☐ **Mentor** ☐ **Scientist** ☐ **Clinician** ☐ **Educator**

Description of Positive Experience*

Save and Resume Later

Submit Form

© 2022 Leitman IM et al. *JAMA Network Open.*

eReferences

1. Appropriate Treatment in Medicine (ATM). A Compendium on Medical Student Mistreatment. A Project of the AAMC Group on Student Affairs. 2000;https://www.aamc.org/media/23541/download Accessed September 6, 2021
2. Medical professionalism in the new millennium: a physician charter. *Ann Intern Med*. Feb 5 2002;136(3):243-6. doi:10.7326/0003-4819-136-3-200202050-00012

© 2022 Leitman IM et al. *JAMA Network Open.*
